# Effect of the Ethanol Extract of Red Okra Pods (*Abelmoschus esculentus* (L.) Moench) to Inhibit Cervical Cancer Cells Growth through Cell Cycle-Associated Oncogenes

**DOI:** 10.1155/2022/1094771

**Published:** 2022-04-26

**Authors:** Nabilatun Nisa, Sri Puji Astuti Wahyuningsih, Win Darmanto, Putut Rakhmad Purnama, Firli Rahmah Primula Dewi, Tipuk Soegiarti, Deya Karsari

**Affiliations:** ^1^Department of Biology, Faculty of Science and Technology, Airlangga University, Surabaya 60115, Indonesia; ^2^Inter-Department of Bioinformatics and Computational Biology, Graduate School, Chulalongkorn University, Pathum Wan, Bangkok 10330, Thailand; ^3^Stem Cell Research and Development Center, Airlangga University, Surabaya 60115, Indonesia

## Abstract

This study aims to evaluate the potency of ethanol extract of red okra pods (EEROP) in inhibiting growth of cervical cancer cells through repression of the cell cycle-associated oncogenes. The EEROP treatment was given to HeLa cells cultured with RPMI medium and incubated at 37°C with 5% CO_2_. The MTT method was used to measure HeLa cell growth and IC_50_ values. The mRNA levels of the three cell cycle-associated oncogenes (*MYC*, *TYMS*, and *MDM2*) were evaluated by qRT-PCR to determine the effect of EEROP treatment on the cell cycle. The lowest percentage of viable cells at 24, 48, and 72 hours after EEROP treatment was in the dose of 1000 *μ*g/mL with a growth percentage of 71.60% at 24 hours, 55.61% at 48 hours, and 46.97% at 72 hours. The IC_50_ values were 2845, 1153, and 776.8 *μ*g/mL for 24, 48, and 72 hours, respectively. The three oncogenes at a dose of 1000 *μ*g/mL significantly decreased the lowest mRNA levels compared to other doses with *MYC* oncogene that experienced the greatest decrease. The mRNA level of dose 1000 *μ*g/mL EEROP at the *MYC* oncogene was 0.014-fold changes, at the *TYMS* oncogene was 0.097-fold changes, and at the *MDM2* oncogene was 0.028-fold changes. The EEROP has been shown to decrease the expression of three cell cycle-associated oncogenes. This is also supported by the growth of HeLa cells that did not increase throughout 24, 48, and 72 hours. However, further research is needed on the main active components in red okra that function as anticancer, so that in the future, okra can not only stop cancer cell growth but also induce cancer cell death.

## 1. Introduction

Cervical cancer has the greatest number of incidences and deaths in women after breast cancer. Cervical cancer also has the most widespread distribution, with cases that occur in almost all parts of the world [1]. It is known that the main cause of cervical cancer is chronic infection with the human papillomavirus (HPV). The HPV virus can integrate its genetic material and cause damage to human DNA and lead to the formation of cancer cells [2]. Secondary factors causing cervical cancer are smoking and alcohol consumption [3]. There are several protective factors such as screening and vaccination, but in lower-middle-income countries, this is still rarely done [4] because of several reasons, such as the lack of education, high costs, and embarrassment of doing this vaccination [5]. Therefore, another alternative is needed to tackle cervical cancer that is more readily accepted and carried out by people in a developing country.

The majority of cancer malignancies are caused by the increased expression of oncogenes in cells, as well as at HeLa cells. The proteins that are encoded by the *MYC* gene trigger the cell cycle and cell proliferation by controlling genes associated with the cell cycle, such as cyclin, CDKs, and the transcription factor E2F [6]. Thymidylate synthase is an enzyme involved in the synthesis of deoxythymidine triphosphate (dTTP). The thymidylate synthase binds to mRNA of c-Myc and p53 and forms the ribonucleoprotein complex that causes a decrease of the p53 protein synthesis [7]. The p53 protein is also strongly associated with the *MDM2* gene. The *MDM2* regulates the p53 protein through a negative feedback mechanism by triggering ubiquitination and degradation of p53 through proteasome [8]. The degradation of p53 protein is mainly regulated by the *MDM2* gene [9]. These three cell cycle-associated oncogenes have been widely studied as markers of tumorigenesis and poor prognosis in several types of cancer, including cervical cancer.

Developing new anticancer drugs is important nowadays because many cancer cells are resistant to currently available drugs [10]. One of the anticancer alternatives that is currently being developed is by nutraceutical. A bunch of common dietary items have appeared as a potential part of the prevention and treatment of cancers and have been considered as a potent strategy [11]. Red okra (*Abelmoschus esculentus* L. Moench) is a vegetable that is widely believed to be a dietary medicine [10]. Okra is a member of the Malvaceae family. The pods of the okra plant are usually eaten when they are not ripe. Okra has antioxidant, antibacterial, antifungal, and anticancer activities [12], with some of the active compounds of okra being from the polyphenol group. Ying et al. [13] showed that polyphenolic compounds found in okra have powerful anticancer activity.

However, there has never been a study evaluating the potency of the ethanolic extract of red okra pods on the growth of HeLa cells and the inhibition of *MYC*, *TYMS*, and *MDM2* oncogenes. This study aims to evaluate the potency of okra extract in inhibiting growth in HeLa cervical cancer cells through cell cycle-associated oncogenes. The polyphenolic content that is captured with EEROP can also be proposed to be a new plant-derived anticancer compound in the future.

## 2. Materials and Methods

### 2.1. Preparation of Ethanol Extract of Red Okra Pod (EEROP)

The ethanol extraction of okra pods was done using the protocols of Yasin et al. [14]. Okra pods obtained from traditional markets were washed and then dried without direct sunlight. Then, the dried okra pods were ground and macerated for 24 hours with absolute ethanol while stirring periodically. The maceration process was repeated three times. The maceration results are then evaporated using a rotary evaporator.

### 2.2. Cell Culture and Treatment Conditions

HeLa cells were obtained from the Stem Cell Research and Development Center Universitas Airlangga. Cells were cultured in RPMI culture medium and incubated at 37°C with 5% CO_2_. For all measurements, an equal volume of FBS-containing medium was used as the control. An ethanol extract of okra pods was given according to the concentration in each group with a DMSO solvent of less than 1%. The dose used in this study was based on Hayaza et al. [15], with modification of wider dose ranges that are 50, 100, 250, 500, and 1000 *μ*g/mL. There were five variations of the EEROP treatment dose used in this study. The group with a dose of 0 *μ*g/mL EEROP became the control group, and the positive control group was given methotrexate (MTX) 10 *μ*g/mL. Cells were then incubated for 24, 48, and 72 hours as studied by Paulraj et al. [16] and then harvested for further tests.

### 2.3. MTT Assay

HeLa cells were divided and cultured in a 96-well plate with 5000 cells per well. After treatment is ended, 15 *μ*L of MTT reagent in 135 *μ*L media was added to each well. Then, it was incubated at 37°C for 4 hours until formazan was formed. Observation of cell conditions was carried out with an inverted microscope. If formazan was formed, a stopper of 50 *μ*L DMSO was added to each well, and the OD value was read at 560 nm and 750 nm.

### 2.4. Determination of the Analyzed Gene

The gene selection was based on references and analysis of microarray data on GEO dataset (GDS) with DataSet Record: GDS3233 (https://www.ncbi.nlm.nih.gov/sites/GDSbrowser?acc=GDS3233). The selection of the GDS3233 dataset is because this dataset has three types of cervical cell samples: normal cells, primary cancer cells, and several types of cancer cell lines including HeLa Cells. The *MYC* gene is an oncogene that has been widely studied. According to the study by García-Gutiérrez [6], *MYC* is the main promoter of the cell cycle. *MDM2* was selected because this oncogene was the major regulator of p53, which is upstream of the CDK1-cyclin A and B protein complex that plays an essential role in the progression of the cell cycle [17–19]. The *TYMS* was chosen because, according to the GDS3233 dataset, it is very highly expressed in both primary cervical cancer cells and cervical cancer cell lines when compared to normal cervical cells.

### 2.5. RNA Extraction and cDNA Synthesis

The cells were seeded into an M6 well plate with 10000 cells per well. After treatment with EEROP in 72 hours, HeLa cells were trypsinized and centrifuged 300 × g for 5 minutes to get the cells pellet. The RNA of HeLa cells was subsequently extracted with a Total RNA Isolation System from Promega based on the manufacturer's protocol. The results of RNA extraction were evaluated with BioDrop at a 260 nm wavelength. The synthesis of cDNA in this study was carried out using the GoScript Reverse Transcription System from Promega using the manufacturer's instruction. PCR for cDNA was performed in the total volume of 20 *μ*L with 5 minutes at 25°C for the priming process, 1 hour at 42°C for the reverse transcription process, and 15 minutes at 70°C for inactivating the reverse transcriptase enzyme.

### 2.6. Real-Time PCR Analysis

Primer designing for three cell cycle-associated oncogenes was carried out in this study (Table 1). The PCR reaction was done at 95°C cycles for 10 minutes to activate the enzyme for one cycle, followed by 45 cycles of denaturation at 95°C for 10 seconds and annealing/extension at 57.5°C for 5 seconds. The threshold value uses the automatic setting of the MyGo Pro application. Measurement of gene expression was carried out by relative quantitative calculations by Livak and Schmittgen [20]. The qPCR result reliability is ensured using melting curve analysis.

### 2.7. Statistical Analysis

All data produced experiments were analyzed with one-way analysis of variance (one-way ANOVA) using IBM SPSS Statistics 25. Data with a significant difference were proceeded with the Duncan test to determine the difference between treatment groups with *α* = 0.05.

## 3. Results

### 3.1. The Effect of EEROP to Inhibit Viability of the HeLa Cells

The MTT test revealed that EEROP treatment could reduce the number of viable cells. The higher percentage of viable cells decreases with the higher dose of EEROP given. In addition, the percentage of viable cells also continued to decrease with the length of incubation time.

**Figure 1:**
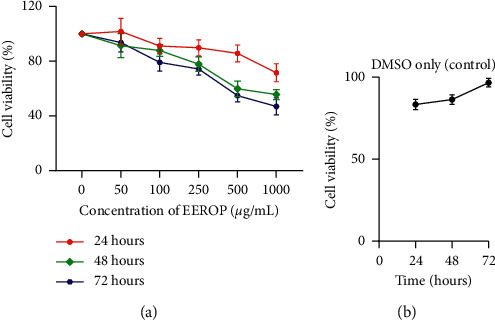
Percentage of cell's viability. (a) EEROP treatment. (b) DMSO only (control).

The results are shown in Figure 1(a), the viable cells in the EEROP treatment were lower at higher EEROP concentrations and longer incubation times. The percentage of viable cells at 24 hours after EEROP treatment at a dose of 50 *μ*g/mL was 101.78%, 100 *μ*g/mL was 91.18%, 250 *μ*g/mL was 89.81%, 500 *μ*g/mL was 85.70%, and 1000 *μ*g/mL was 71.60%. At the incubation time of 48 hours, the percentage of viable cells after treatment of EEROP at a dose of 50 *μ*g/mL was 91.40%, 100 *μ*g/mL was 87.89%, 250 *μ*g/mL was 77.88%, 500 *μ*g/mL was 59.85%, and 1000 *μ*g/mL was 55.61%. While, the percentage of viable cells after EEROP administration with 72 hours incubation time at 50 *μ*g/mL was 93.65%, 100 *μ*g/mL was 79.12%, 250 *μ*g/mL was 74.33%, 500 *μ*g/mL was 54.81%, and 1000 was 46.97%. This result is the opposite of Figure 1(b), which shows that the percentage of viable cells increases over time.

**Figure 2:**
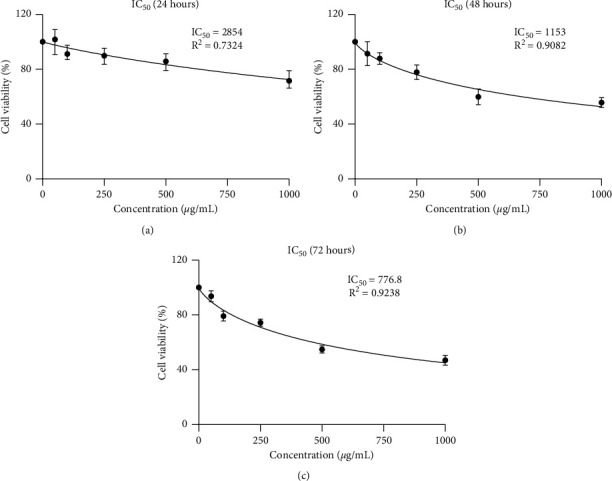
The IC_50_ value of ethanol extract of red okra pods (EEROP) to HeLa cells, evaluated after (a) 24 hours, (b) 48 hours, and (c) 72 hours.

Based on the percentage of viable cells, we arrange the graphic of IC_50_ EEROP against the HeLa cells in both concentration- and time-dependent manner. From Figure 2, it was found that the IC_50_ value continued to decrease with incubation time, but the value was still relatively high. The group with 1000 *μ*g/mL of EEROP had the lowest percentage of viable cells at all incubation times compared to other doses. Although this treatment has not induced HeLa cells to massive cell death, EEROP treatment can significantly inhibit the growth of HeLa cells.

### 3.2. Repression of Cell Cycle-Associated Oncogenes

**Figure 3:**
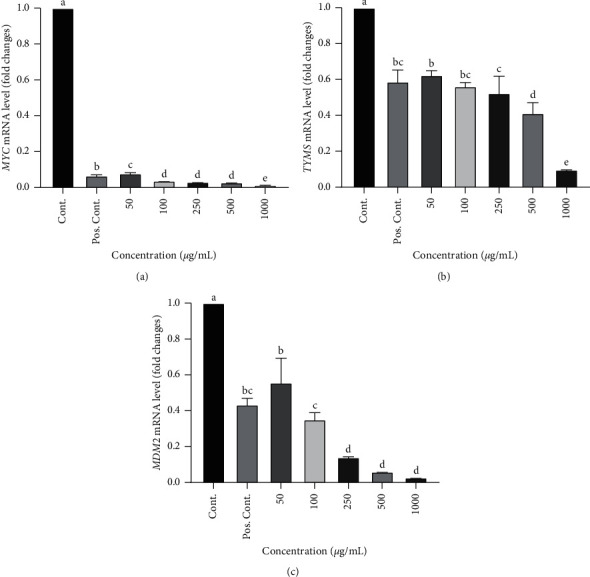
The mRNA levels of cell cycle-associated oncogenes. Different superscript letters indicate significant differences (*P* < 0.05) according to Duncan's multiple range test.

Expression values are reported as the mRNA levels with normalized control group expression. Experiments were performed in triplicates, and results were compared with one housekeeping gene (*ACTB*). Error bars depict the mean ± S.E.M. The mRNA levels of the *MYC* oncogene were significantly decreased after treatment with EEROP, as shown in Figure 3(a). The dose of 1000 *μ*g/mL had the lowest mRNA levels with a decrease of 6.154-fold changes. The decrease in *MYC* mRNA levels at a dose of 1000 *μ*g/mL was also significant compared to the positive control group, which experienced mRNA levels of 0.066. Meanwhile, mRNA levels for doses of 50, 100, 250, and 500 *μ*g/mL were 0.078, 0.037, 0.031, and 0.027-fold changes, respectively. In general, the *MYC* oncogene decreased the most when compared to the other two oncogenes, and this was due to its prominent role in the cell growth process.

From Figure 3(b), it is known that the dose of EEROP 1000 *μ*g/mL was also shown to have the most significant decrease in *TYMS* oncogene's mRNA levels compared to the whole group, which was 0.097-fold changes. Positive control only reduced the mRNA levels of *TYMS* oncogene which is 0.587-fold changes, while the mRNA levels for EEROP doses of 50, 100, 250, and 500 *μ*g/mL were 0.624, 0.562, 0.524, and 0.412-fold changes, respectively. However, when compared to the other two oncogenes, *TYMS* had the lowest decreased expression.

In line with the other two oncogenes, the EEROP treatment can reduce the mRNA levels of the oncogene *MDM2*, and the dose of 1000 *μ*g/mL also experienced the most significant decrease of the mRNA levels compared to other doses. Based on Figure 3(c), the positive control group only has the mRNA levels of 0.435, and the mRNA levels of EEROP doses of 50, 100, 250, 500, and 1000 *μ*g/mL were 0.557, 0.351, 0.140, 0.059, and 0.028-fold changes, respectively.

## 4. Discussion

The potency of ethanol extract of red okra pods (EEROP) as an anticancer, especially cervical cancer, was evaluated in this study in vitro. The HeLa cells are the most widely used in vitro models for cervical cancer. The EEROP did not lead to the massive death of HeLa cells. However, EEROP treatment significantly inhibited the growth of HeLa cells and suppressed the mRNA levels of the three cell cycle-associated oncogenes.

The 1000 *μ*g/mL group of EEROP, which is the highest dose, had the lowest cell growth that was evidenced by a decrease in the number of viable cells from 24, 48, and 72hours. The lowest mRNA levels of all oncogenes were also experienced in this group. This shows that EEROP has potential as an anticancer agent. Although the lowest viability and the lowest oncogene expression were achieved by the highest dose group, the lower dose group was able to significantly reduce the growth of HeLa cells. The incubation at 72 hours showed that the IC_50_ value was 776.8 *μ*g/mL, which is below 1000 *μ*g/mL (the highest dose). As well as with oncogene expression, the decrease in *MYC* expression at 100 *μ*g/mL, in *TYMS* at 500 *μ*g/mL, and in *MDM2* at 250 *μ*g/mL was significantly lower than MTX as a commercial cancer drug. Therefore, further research on the optimal dose of EEROP as an anticancer can be studied more deeply in the future.

Evaluation of oncogene expression is essential in eukaryotes because protein expression is not the same as a gene but becomes mRNA before being expressed. Oncogenes expressed in HeLa cells are so high that HeLa cells can maintain their viability without being affected by aging. Just like cervical cancer in general, the genome of HeLa cells is also integrated with genes from the HPV virus. The genes in HeLa cells most affected by this integration are the *MYC* genes that encode the c-Myc protein [21].

Ying et al. [13] revealed that the main antiproliferative components of okra are carolignan and 4′-hydroxy phenethyl trans-ferulate. However, the mechanism of this component as an anticancer is still not widely studied. A study by Arapitsas [22] revealed that the most abundant phenolic components in okra were hydroxycinnamic acid and quercetin. A recent study by Li and Hu [23] showed that hydroxycinnamic acid has been shown to decrease c-Myc expression, leading cancer cells to die. In line with this, Gao et al. [24] revealed that ferulic acid, which is a derivative of hydroxycinnamic acid, can trigger HeLa cells to lead to cell cycle arrest; this is indicated by decreased expression of cyclin-D1 and p53 and p21 proteins, where cyclin-D1 expression is controlled by c-Myc protein and p53 and p21 are controlled by *MDM2*. A study conducted by Chen et al. [25]showed that the derivative component of hydroxycinnamic acid can inhibit histone deacetylase and inosine monophosphate which makes cancer cells enter cell cycle arrest and leads to apoptosis. The PI3K/Akt/mTOR pathway is also an important signalling pathway in the regulation of the cell cycle, promoting tumour cell survival and proliferation in many cancers [26]. Cell cycle inhibition at this pathway can be inhibited by hydroxycinnamic acid combined with *β*-carbolines [27].

Quercetin can inhibit the expression of c-Myc, where c-Myc is a transcription factor of various genes related to cell growth. Two studies that have been conducted on cervical cancer have shown that the decreased c-Myc expression due to quercetin can exert a cascade effect and lead to inhibition of the proliferation of cervical cancer cells, including inhibiting RPS19 and epithelial-to-mesenchymal transition (EMT) signalling [28] and RPS12 [29], which causes a decrease in cancer cell proliferation. Quercetin also plays a role in reducing *MDM2* expression indirectly through stimulation of the expression of the CDKN2A gene. The product of CDKN2A can interact with *MDM2* to decrease its expression, so that p53 protein production can be increased [30]. We guess that the important and interrelated roles of *MYC* and *MDM2* made those genes have the most decreased expression due to the EEROP treatment.

The EROP treatment can significantly reduce *MYC* expression at the 100–1000 *μ*g/mL dose compared to the positive control. In MDM2, also compared with the positive control group, the results of 50 and 100 *μ*g/mL EEROP treatment were not significantly different, while the groups with doses of 250–1000 *μ*g/mL can more significantly decrease the *MDM2* expression. This shows that EEROP has a strong potential to have anticancer performance like commercial drugs.

*TYMS* experienced the least of expression compared to the other two oncogenes, presumably due to its role in the cell cycle. The current commonly revealed *TYMS* role is related only in the synthesis of thymine. Although *TYMS* plays an important role in the cell cycle, its involvement is not influenced or affected by many pathways such as *MYC* or *MDM2*. Despite the level of *TYMS* expression not being suppressed as high as the other two oncogenes, the most decreased *TYMS* expression was also experienced in the group with the highest dose. The smallest dose of EEROP treatment was not significantly different from the positive control, and the 500 *μ*g/mL group significantly suppressed *TYMS* expression more than MTX as a commercial anticancer drug.

In summary, the EEROP treatment can inhibit the growth of HeLa cells by decreasing viability over incubation times and by decreasing the expression of three cell cycle-related oncogenes. This can be the beginning for further research to reveal more about the precise bioactive components of red okra as an anticancer, determining the optimal dose of use and detailing the mechanism of red okra as an anticancer.

## 5. Conclusions

The ethanol extract of red okra pods (EEROP) has been shown to decrease the expression of three oncogenes in cervical cancer cells associated with the cell cycle. These are also supported by the growth of HeLa cells that did not increase over 24, 48, and 72hours. The group with the lowest cell viability and the most decreased oncogene expression was at a dose of 1000 *μ*g/mL.

## Figures and Tables

**Table 1:** Primer sequences.

Gene	Sequences
*ACTB*	F: CCACACTGTGCCCATCTACG
	R: AGGATCTTCATGAGGTAGTCAGTCAG

*MYC*	F: GGAGGAACAAGAAGATGAGG
	R: GTAGTTGTGCTGATGTGTGG

*TYMS*	F: GAACCCAGACCTTTCCCAAA
	R: AACTTTTACCTCGGCATCCA

*MDM2*	F: GGGCTTTGATGTTCCTGATT
	R: CTTGGGTTTCTTCCCTTTCA

## Data Availability

The data used to support the findings of this study are available from the corresponding author upon request.
